# P183: A multi-pronged approach to unravel rhinovirus transmission in a children’s hospital: towards effective infection control

**DOI:** 10.1186/2047-2994-2-S1-P183

**Published:** 2013-06-20

**Authors:** J Rahamat-Langendoen, M Ciccolini, E Schölvinck, A Friedrich, H Niesters

**Affiliations:** 1 Department of Medical Microbiology, Division of Clinical Virology, Groningen, the Netherlands; 2 Department of Medical Microbiology, Division of Infectious Disease Epidemiology, Groningen, the Netherlands; 3 Beatrix Children’s Hospital, University of Groningen, UMC Groningen, Groningen, the Netherlands

## Introduction

Human rhinovirus (HRV) is associated with serious respiratory illness, particularly in patients with pulmonary comorbidities. Little is known about nosocomial transmission of HRV. A good understanding of the hospital epidemiology of HRV is required to evaluate the effect of infection control strategies.

## Methods

We employed a comprehensive approach including classical and molecular epidemiology, as well as mathematical modeling methods. Data on HRV detection, clinical symptoms and infection control measures were retrieved from a prospective project into respiratory infections in hospitalized children between October 2009 and January 2011. HRVs were characterized using sequence analysis of the VP4/VP2 genes. An agent-based, multi-ward, stochastic mathematical model was developed to study the impact of infection control measures on the prevalence of HRV positive patients.

## Results

254 HRV disease episodes from 162 patients were included. Based on first day of illness, 69 episodes (27%) were hospital acquired. Infection control measures were implemented one day after start of illness (median value). Using phylogenetic analysis, eight clusters of patients were identified, where epidemiological data suggested transmission within the same ward. The model was used to assess the influence of variations in time-to-start and duration of control measures, changes in admission rate of HRV positive cases as well in the rate of visitor mediated transmission on the effectiveness of infection control measures.

## Conclusion

Nosocomial HRV infections occur more frequently than expected. The effectiveness of infection control strategies depends on a complex set of interrelated factors. Combining epidemiological methods, sequence based information and mathematical modeling techniques leads to valuable information on our understanding of nosocomial transmission dynamics, which contributes to the implementation of appropriate infection control interventions. This can serve as a model for identifying intervention points for transmission of respiratory microorganisms in general.

## Disclosure of interest

None declared

